# Uridine Metabolism in HIV-1-Infected Patients: Effect of Infection, of Antiretroviral Therapy and of HIV-1/ART-Associated Lipodystrophy Syndrome

**DOI:** 10.1371/journal.pone.0013896

**Published:** 2010-11-15

**Authors:** Pere Domingo, Javier Torres-Torronteras, Virginia Pomar, Marta Giralt, Joan Carles Domingo, Maria del Mar Gutierrez, José M. Gallego-Escuredo, Maria Gracia Mateo, Pedro Cano-Soldado, Irene Fernandez, Marçal Pastor-Anglada, Francesc Vidal, Francesc Villarroya, Antoni Andreu, Ramon Marti

**Affiliations:** 1 Infectious Diseases Unit, Hospital de la Santa Creu I Sant Pau, Barcelona, Spain; 2 Laboratory of Mitochondrial Disorders, Institut de Recerca Hospital Vall d'Hebron and CIBERER, Barcelona, Spain; 3 Departament de Bioquimica i Biologia Molecular and Institut de Biomedicina de la Universitat de Barcelona (IBUB), Barcelona, Spain; 4 Infectious Diseases Unit, Hospital Universitari Joan XXIII, Tarragona, Spain; University of Cape Town, South Africa

## Abstract

**Background:**

Uridine has been advocated for the treatment of HIV-1/HAART-associated lipodystrophy (HALS), although its metabolism in HIV-1-infected patients is poorly understood.

**Methods:**

Plasma uridine concentrations were measured in 35 controls and 221 HIV-1-infected patients and fat uridine in 15 controls and 19 patients. The diagnosis of HALS was performed following the criteria of the Lipodystrophy Severity Grading Scale. Uridine was measured by a binary gradient-elution HPLC method. Analysis of genes encoding uridine metabolizing enzymes in fat was performed with TaqMan RT-PCR.

**Results:**

Median plasma uridine concentrations for HIV-1-infected patients were 3.80 µmol/l (interquartile range: 1.60), and for controls 4.60 µmol/l (IQR: 1.8) (P = 0.0009). In fat, they were of 6.0 (3.67), and 2.8 (4.65) nmol/mg of protein, respectively (P = 0.0118). Patients with a mixed HALS form had a median plasma uridine level of 4.0 (IC95%: 3.40–4.80) whereas in those with isolated lipoatrophy it was 3.25 (2.55–4.15) µmol/l/l (P = 0.0066). The expression of uridine cytidine kinase and uridine phosphorylase genes was significantly decreased in all groups of patients with respect to controls. A higher expression of the mRNAs for concentrative nucleoside transporters was found in HIV-1-infected patients with respect to healthy controls.

**Conclusions:**

HIV-1 infection is associated with a decrease in plasma uridine and a shift of uridine to the adipose tissue compartment. Antiretroviral therapy was not associated with plasma uridine concentrations, but pure lipoatrophic HALS was associated with significantly lower plasma uridine concentrations.

## Introduction

**Figure 1:**
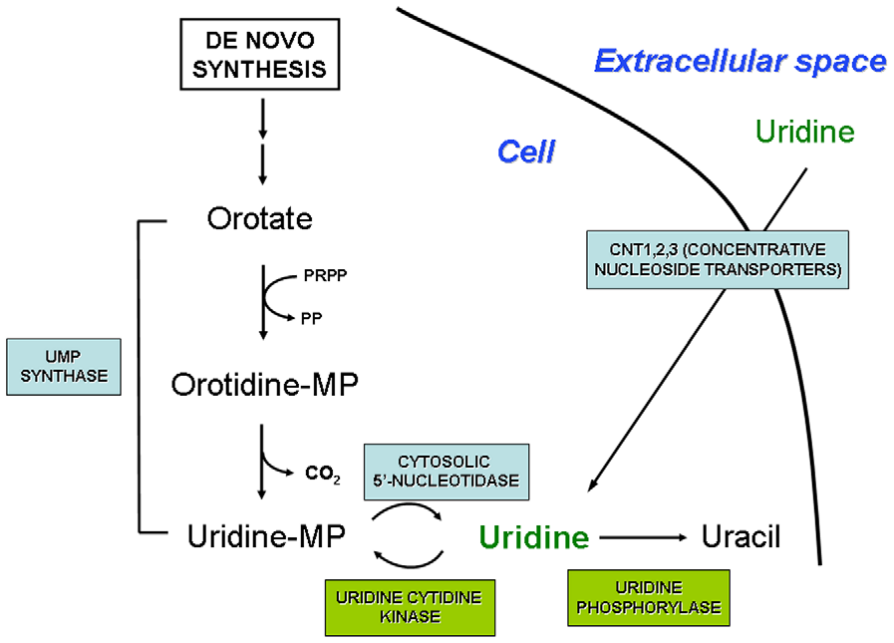
Schematic representation of uridine synthesis and its transport into the cell. MP  =  monophosphate.

Pyrimidines are synthesized *de novo* through a multistep process starting from glutamine and carbon dioxide to form the pyrimidine ring, orotic acid [1]. The synthesis of orotic acid is catalyzed by dihydroorotate dehydrogenase (DHODH), an enzyme located in the inner mitochondrial membrane, and functional connection to the respiratory chain via ubiquinone ensures efficient oxidation of dihydroorotate [2]. Then, orotate is converted to its nucleotide form in the presence of 5-phosphorylribose-pyrophosphate. Orotate monophosphate is converted by a multifunctional enzyme, uridine monophosphate (UMP) synthase to the nucleotide UMP (Figure 1). UMP is the pivotal nucleotide from which uridine nucleotides di-and triphosphates are formed by ATP-dependent kinases [3]. A large portion of the pyrimidines are salvaged from the degradation of the nucleic acids and nucleotides [4]. The concentration of circulating plasma uridine is tightly regulated throughout different species and individuals [5], [6]. The liver appears to have this homeostatic control on uridine degradation and formation [7]. Uridine is essentially cleared in a single pass through the liver and is replaced by “new uridine” formed by *de novo* synthesis [7].

Besides the critical role of uridine in the synthesis of RNA and bio-membranes, there is a role for uridine in regulating a series of biological functions such as complex effects in the regulation of vascular resistance, a role in spermatogenesis, a modulatory effect on the peripheral nervous system, and maintenance of normal CNS activity [8]. In animal models, uridine reduces anxiety, is a sleep-inducing factor, and may regulate body temperature [8]. Aside from its physiological effects, uridine appears to have remarkable functions in tissues under stress or pathological situations such as restoration of myocardial ATP concentrations [9], maintenance of brain metabolism during ischemia or severe hypoglycemia [10], recovery from neuronal degeneration produced by diabetic neuropathy [11], and as a rescue agent in 5-fluorouracil therapy to decrease bone marrow and gastrointestinal toxicity [12].

The triphosphates are the active forms of the nucleoside analogue reverse transcriptase inhibitors, but they also inhibit polymerase gamma, resulting in mitochondrial DNA depletion [13], although reduction of mitochondrial DNA copy number is also associated to HIV infection in itself [14]. Because the de novo pyrimidine synthesis needs the correct function of the mitochondrial respiratory chain, pyrimidine nucleotide depletion has been hypothesized to contribute to this toxicity by exacerbating the competitive effect of the analogues [15]. However, the effect of these drugs on patients' uridine concentrations has been studied in few cases [16]. Uridine has been able to abrogate the adverse effects of antiretroviral pyrimidine analogues in adipocytes [17], and the benefitial effects of uridine supplementation in cell culture models of stavudine toxicity seems to involve the recovery from nucleoside analogue-induced pyrimidine depletion, because uridine supplementation also prevented the effects of redoxal, a specific DHODH inhibitor which, in turn, exacerbated the mitochondrial toxicity of the stavudine [15]. In a clinical trial, supplementation of uridine to patients on thymidine analogues was associated with fat recovery in limbs [18].

Our working hypothesis was that variations in plasma and fat concentrations of uridine may be involved in the modulation of the toxic effects of thymidine analogues. Specifically, we postulated that the toxic effects of thymidine analogues might result in pyrimidine depletion, detectable as a reduction of systemic and/or fat uridine concentrations. Therefore, the aim of the present study was to look for a relationship between plasma and fat uridine concentrations and HIV-1/highly active antiretroviral (HAART)-associated lipodystrophy syndrome (HALS).

## Materials and Methods

### Subjects

All patients were recruited through the same clinic at the *Hospital de la Santa Creu I Sant Pau*, which attends a population of 1455 HIV-1-infected patients on active follow-up, and were consecutive patients with an established diagnosis of HIV-1 infection. Patients were eligible whether or not they had HALS, whether or not they were on antiretroviral therapy, and if they were not taking uridine or derivatives. Subjects who were hospitalized or had a frank cognitive impairment such as delirium or dementia on enrolment were not eligible. Patients with opportunistic infections, acute hepatitis, liver insufficiency, neoplasms or fever of undetermined origin were excluded from the study. At the time of study entry no patient used any other drug known to influence glucose metabolism or fat distribution. Informed consent was obtained from the patients at study entry. The diagnosis of AIDS was based on the 1993 revised case definition of the Centers for Disease Control and Prevention (CDC) [19]. Controls were recruited among Hospital personnel and had to be negative for HIV-1 infection and be between 35 and 45 years of age with a proportion of males of about 70%. To be eligible they did not have to meet any of the exclusion criteria. Written informed consent was obtained from patients and controls at study entry. The study was approved by the Ethics Committee of the *Hospital de la Santa Creu i Sant Pau*.

### Body composition measurements, definitions of HALS and metabolic syndrome, and biochemistry laboratory measurements

Body composition measurements, definitions of HALS and metabolic syndrome have been described elsewhere [20]. All laboratory investigations were performed as previously described [21]. All laboratory samples, including those for uridine determination, were obtained after a 12 hr overnight fasting. (Supporting information file S1).

### Fat tissue samples

These were obtained from subcutaneous adipose tissue (SAT) depots through a small surgical biopsy performed by an 8 mm punch under local anesthesia with mepivacaine. One half of the SAT obtained was immediately frozen in liquid nitrogen and stored at −80°C until RNA extraction (see below). The remaining was used in fresh for determining fat uridine concentrations.

### Measurement of plasma and fat uridine concentrations

For uridine determination, anticoagulated blood was centrifuged at 1,500× g and plasma was separated and kept at −20 C until analysis. Fat tissue was homogenated in the presence of 10 volumes (e.g., 50 mg of tissue +500 µl) of homogenization buffer (10 mmol/l HEPES pH 7.5; 5 mmol/l EDTA; 5 mmol/l DTT; 5 mmol/l MgCl_2_; plus *Complete-Mini™* protease inhibitor cocktail, Roche), in an ice-cold bath. After centrifugation, the supernatant was saved and protein concentration was determined by the Bradford method [22].

Uridine was measured by a binary gradient-elution HPLC method. 200 µl of plasma or tissue homogenate were deproteinized by the addition of 9 µl of cold perchloric acid 11.7 mol/l (final concentration 0.5 mol/l) and kept 5 min on ice. Precipitated proteins were eliminated by centrifugation and 5 µl of the supernatant were injected into an Acquity UPLC apparatus (Waters, Milford, MA) and eluted at 0.5 ml/min with a saline buffer eluent A (20 mM ammonium acetate, pH 5.6) and an organic eluent B (methanol gradient grade) according to the following gradient: 0 to 1.1 min, 100% eluent A; 1.1 to 6.1 min, 100% to 82.6% eluent A; 6.1 to 6.2 min, 82.6 to 100% eluent A; 6.2 to 7.2 min, 100% eluent A. The column used was an Acquity UPLC BEH C18 column 100×2.1 mm, 130 Å pore size, 1.7 µm particle size (Waters). Optical absorbance of the eluate was monitored at 267 nm and definitive identification of the uridine peak was based upon retention time and treatment of a second aliquot of the sample with a large excess of purified *E. coli* thymidine phosphorylase (Sigma-Aldrich, St. Louis, MO) to eliminate enzymatically the uridine peak. The area from residual peaks coeluting with uridine and still present after treatment with *E. coli* thymidine phosphorylase was substracted from the area of the uridine peak obtained in the untreated aliquot, to ensure no overestimation of uridine concentration. The quantitation of the nucleoside was based on peak areas using external aqueous standards. The method had a between-day imprecision of 2.3% (coefficient of variation) for a uridine concentration of 4.30 µmol/l. Uridine concentrations in plasma of ∼3 to 5 µmol/l are found in different species and individuals [5], [6], [12].

### Expression of genes encoding uridine metabolism-related enzymes and nucleoside transporters

RNA extraction was performed using a column-affinity based methodology (Rneasy, Qiagen, Hilden. Germany). On-column DNA digestion was performed during RNA purification (Rnase-Free Dnase set, Qiagen). TaqMan Reverse Transcription and RT-PCR reagents were used for mRNA analysis (Applied Biosystems, Foster City, USA). One microgram RNA was transcribed into cDNA random-hexamer primers and the real-time reverse transcriptase-polymerase chain reaction was performed on the ABI PRISM 7700HT sequence detection system (Applied Biosystems). The TaqMan RT-PCR reaction was performed in a final volume of 25 µl using TaqMan Universal PCR Master Mix, NoAmpErase UNG reagent and the specific gene expression primer probes. The TaqManGene Expression assays (Applied Biosystems) used were: uridine-monophosphate synthase, Hs00923516; uridine cytidine kinase-1, Hs00258815; uridine phosphorylase, Hs00427695; uridine 5′-monophosphate hydrolase (also called cytosolic 5′-nucleotidase III), Hs00826433; concentrative nucleoside transport (CNT-1), Hs00984402; CNT-2, Hs01035852; CNT-3, Hs00223220. Controls with no RNA, primers, or reverse transcriptase were included in each set of experiments. Each sample was run in duplicate and the amount of the mRNA for the gene of interest in each sample was normalized to that of the reference housekeeping control (HPRT1, Hs999999009), as already reported [23] using the comparative (2-ΔCT) method. Calculations based on a second, independent, housekeeping gene (18S rRNA) led to similar results. Data are expressed as means ± SEM.

### Measurement of plasma fatty acid concentrations

The serum composition of fatty acids was determined using the method by Lepage and Roy [24]. Aliquots of 300 µL of plasma were transferred into glass tubes for direct transesterification. 2 mL of methanol-benzene (4∶1, v/v) with internal standard (heptadecanoic acid, C17:0) and 0.01% butylhydroxytoluene, as antioxidant. Samples were vortexed at low speed while slowly adding 200 µL of acetyl chloride, little by little, over a period of 2 minutes. The tubes were tightly closed with teflon-lined caps and vortexed 30 seconds.

Samples were then heated for 60 minutes at 100°C in a heating block and shaking continuously at 600 rpm. After the tubes had been cooled to room temperature, five millilitres of 6% (w/v) potassium carbonate were then added. The samples were vortexed for 30 seconds and centrifuged at 2500 rpm for 20 minutes at 15°C. The fatty acid methyl esters contained in the upper benzene phase were transferred to gas chromatography vials and stored at 4°C until injection into the chromatograph.

The analysis was performed on a Varian CP-3900 gas chromatograph equipped with a flame ionization detector, using a capillary column model CP9205-VF-WAXms (Varian), 30 m length ×0.25 mm internal diameter ×0.25 µm film thickness. Individual fatty acids were identified by order of elution and upon comparison with known commercially prepared fatty acid standards (GLC 566-C, Nu-Chek Prep Inc.). Fatty acid methyl ester peaks were identified by comparison of retention times of standards and quantified in comparison to known commercially prepared reference standards. The percentage of each fatty acid class was expressed as percentage of total fatty acids.

### Statistical analyses

All analyses were performed with the Statistical Package for Social Sciences version 17.0 (SPSS, Chicago, IL). Data are expressed as mean ± SD or median with interquartile range (IQR). Data that were not normally distributed, as determined using the Kolmogorov-Smirnov test, were logarithmically transformed before analysis. Student's t test was used for comparison between two groups, Pearson's correlations or one-way ANOVA and multiple testing were corrected using Bonferroni correction. Stepwise logistic regression analysis was used to examine the association of plasma uridine concentrations and other parameters with HALS. The variables selected to enter into stepwise regression were those that correlated significantly with plasma uridine concentrations (after Bonferroni correction for multiple testing). In all statistical tests, P values <0.05 were considered significant.

## Results

### Population studied

Two-hundred and twenty one HIV-1-infected patients and 35 healthy controls were studied. The demographics, HIV-1 infection, and antiretroviral exposure parameters from patients and controls are shown in table 1. Mean duration of HIV-1 infection was 7.8±6.8 years (median: 7.0 [IQR: 12.2 years]), and 63 patients (28.5%) had had a prior AIDS-defining condition. Fifty-nine patients were co-infected with hepatitis C virus (26.7%), whereas 10 (4.5%) had chronic hepatitis B virus infection. Anthropometric, metabolic data and plasma uridine concentrations are shown in Table 2.

**Table 1:** Demographics, HIV-1 infection and antiretroviral exposure parameters.

Parameter	Controls(n = 35)	HIV-1-infected patients			P value
		Naïve(n = 78)	HALS-(n = 59)	HALS+(n = 84)	
Age, mean ± SD, (y)	43.7±5.0	36.9±10.2	44.1±8.0	46.2±8.9	< 0.0001
Sex, men (%)	25 (71.4)	67 (85.9)	46 (77.9)	60 (71.4)	0.1302
Years of infection	----	2.0 (1.0)	11.0 (10.0)	13.0 (6.0)	< 0.0001
AIDS (%)	----	3 (3.9)	18 (30.6	42 (50.0)	< 0.0001
HCV infection (%)	----	7 (9.5)	16 (27.6)	36 (42.9)	< 0.0001
CD4 count/mm^3^	----	431 (302)	566 (416)	50 (463)	0.0136
Plasma viral load (log_10_)	----	4.3 (1.2)	1.8 (1.1)	1.7 (1.5)	< 0.0001
**Antiretroviral drug exposure**					
NRTI (m)	----	----	190.0 (106.0)	225.0 (83.0)	0.0069
NNRTI (m)	----	----	35.0 (52.7)	49.0 (48.5)	0.0519
PI (m)	----	----	43.0 (51.2)	49.0 (48.0	0.1114
AZT (m)	----	----	22.0 (71.0)	20.0 (47.7)	0.5533
AZT (g)	----	----	297.0 (852.0)	240.0 (824.5)	0.7448
d4T (m)	----	----	25.0 (61.2)	64.0 (37.5)	< 0.0001
d4T (g)	----	----	62.4 (138.9)	144.9 (89.7)	< 0.0001
3TC/FTC (m)	----	----	50.0 (77.0)	52.0 (65.5)	0.5220
ddI (m)	----	----	10.5 (33.5)	35.5 (61.5)	0.0028
ABC (m)	----	----	0(0)	0 (14.0)	0.8825
TDF (m)	----	----	10.0 (35.0)	8.0 (34.5)	0.6102
EFV (m)	----	----	0 (35.0)	2.5 (44.0)	0.2175
NVP (m)	----	----	0 (33.5)	6.0 (46.0)	0.1400

Parameters are expressed as median (interquartile range) unless specified. HCV, hepatitis C virus, NRTI  =  nucleoside-analogue reverse transcriptase inhibitor, NNRTI  =  non nucleoside-analogue reverse transcriptase inhibitor; PI  =  protease inhibitor; AZT  =  zidovudine, d4T  =  stavudine, 3TC  =  lamivudine, FTC  =  emtricitabine, ddI  =  didanosine, ABC  =  abacavir, TDF  =  tenofovir; EFV  =  efavirenz, NVP  =  nevirapine, m  =  months; g  =  grams. HALS  =  HIV-1/HAART-associated lipodystrophy syndrome.

**Table 2:** Anthropometric, metabolic data and plasma uridine levels.

Parameter	Controls(n = 35)	HIV-1-infected patients			P value
		Naïve(n = 78)	HALS-(n = 59)	HALS+(n = 84)	
BMI	24.4 (2.7)	23.6 (4,6)	23.8 (5.2)	23.5 (4.7)	0.1307
Waist circumference (cm)	89.0 (13.2)	86.0 (14.0)	88.0 (13.0)	86.5 (15.5)	0.0055
Waist-to-hip ratio	0.88 (0.09)	0.91 (0.11)	0.93 (0.09)	0.93 (0.11)	< 0.0001
Total body fat (%)	24.8 (5.8)	21.5 (9.9)	22.5 (10,2)	19.7 (10.5)	< 0.0001
Trunk/apendicular fat ratio	1.15 (0,5)	1.4 (1.2)	1.4 (1.1)	2.4 (1.2)	< 0.0001
Total cholesterol (mmol/l)	5.2 (1.4)	4.7 (1.5)	4.9 (1.0)	4.8 (1.7)	0.0003
Triglycerides (mmol/l)	0.82 (0.43)	1.5 (1.4)	1.9 (1.3)	2.0 (1.7)	< 0.0001
HDL cholesterol (mmol/l)	1.5 (0.5)	1.2 (0.5)	1.2 (0.5)	1.2 (0.4)	< 0.0001
LDL cholesterol (mmol/l)	3.3 (1.3)	2.7 (1.2)	2.5 (1.0)	2.8 (1.0))	0.0027
MUFAs (% with respect to total fatty acids)	23.9 (4.3)	26.3 (5.0)	27.9 (6.6)	28.3 (5.9)	< 0.0001
PUFAs (% with respect to total fatty acids)	44.7 (4.0)	40.4 (5.5)	38.7 (7.3)	37.2 (7.6)	< 0.0001
Glucose (mmol/l)	4.8 (0.6)	5.1 (0,9)	5.3 (0,7)	5.5. (1.1)	< 0.0001
Insulin (pmol/l)	26.0 (33.2)	48.5 (63.5)	48.0 (54.2)	85.5 (61.5)	< 0.0001
HOMA-IR	0.5 (0.7)	0.9 (1.1)	0.9 (1.0)	1.6 (1.2)	< 0.0001
Systolic BP (mm Hg)	118.0 (17.7)	120 (16.5)	120 (20.0)	120 (20.0)	0.2649
Diastolic BP (mm Hg)	69.0 (15.0)	75.0 (10.0)	78 (12.0)	78 (10.0)	0.0081
Metabolic syndrome (%)	5 (14.3)	5 (6.4)	18 (30.5)	28 (33.3)	< 0.0001
Uridine (µmol/l)	4.6 (1.8)	3.8 (1.3)	3.8 (1.6)	3.7 (1.7)	0.0034

Parameters are expressed as median (interquartile range) unless specified. P-values between individual groups are shown when <0.05. BMI, body mass index; HDL, high density lipoprotein; LDL, low density lipoprotein, HOMA-IR, homeostasis model assessment of insulin resistance; HALS  =  HIV-1/HAART-associated lipodystrophy syndrome.

### Antiretroviral drug exposure and immuno-virological situation

Among treated patients, 59 (41.3%) had undetectable viral load at the moment of the study. The other 84 treated patients presented a median detectable viral load of 2.3 log_10_ copies/ml (IQR: 1.1 log_10_ copies/ml). The mean CD4 count was 589±320 cells/mm^3^ (median: 531 [IQR. 425] cells/mm^3^). Nadir CD4 cell count was <100 cells/mm^3^ in 57 patients (25.8%). The cumulated exposure to antiretroviral drugs is shown in table 1. Twenty patients (13.9%) of the treated group were on an antiretroviral regime that included AZT whereas 25 (17.5%) were on a d4T-based regime at the moment plasma and fat uridine concentrations were measured. There were not statistically significant differences in plasma and fat uridine concentrations between patients currently on AZT (P = 0.9930) or d4T (P = 0.4944), either considered individually or as a combined group (P = 0.5809).

### Diagnosis of HALS and metabolic syndrome

HALS, based on 7 or more points on the Lipodystrophy Scale Grading Score (LSGS), was diagnosed in 84 treated patients (58.7%) whereas absence of HALS was found in 59 treated patients (41.3%), and in all the naives. All 84 patients presented with lipoatrophy, but in addition 44 (52.4%) had a mixed lipoatrophic-lipohypertrophic form. Differences between HALS and non-HALS patients are shown in tables 1 and 2. Patients with a mixed form had a median plasma uridine level of 4.0 (IC95%: 3.40–4.80) whereas in those with isolated lipoatrophy it was 3.25 (2.55–4.15) µmol/l/l (P = 0.0066).

### Plasma uridine concentrations and its correlation with cardio-metabolic, HIV-1 infection and antiretroviral therapy factors

Plasma uridine concentrations for HIV-1-infected patients ranged from 1.60 to 7.4 µmol/l/l, with a mean value of 3.85±1.18 µmol/l (median: 3.80; IQR: 1.60). The corresponding values for controls were: range: 2.0–9.4 µmol/l, mean: 4.36±1.28 µmol/l, median: 4.60, IQR: 1.80 µmol/l (P = 0.0009). Plasma uridine concentrations were not significantly different between HALS and non-HALS patients (P = 0.3377). Patients with plasma uridine concentrations <3 µmol/l were 8.9% among controls, 16.7% among naïves, 19.0% among treated patients without HALS, and 29.6% among patients with HALS (P = 0.0460). There was no sex difference in plasma uridine concentrations for HIV-1-infected patients, but there were statistically significant differences between controls (men: median 5.0 [IQR: 1.5] vs. women: 3.45 [1.4], P = 0.0262). In naïve patients, there was a negative correlation between plasma concentrations of uridine and viral load (R = −0.2580, P = 0.0228). Patients who were currently receiving treatment with AZT or d4T had a median plasma uridine level of 3.90 [IQR: 1.67] whereas in those who did not it was 3.70 [IQR: 1.80) µmol/l (P = 0.4278). There was no correlation between cumulated d4T use either in time (months) (R = 0.094, P = 0.3935) or grams (R = 0.078, P = 0.4815). There were not statistically significant differences between patients who did or did not have AIDS, CD4 nadir <100 cells/mm^3^, did or did not have not clinical toxicity (peripheral neuropathy or pancreatitis), HALS, HCV infection, and metabolic syndrome with respect to plasma uridine concentrations. Lean subjects had median plasma uridine concentrations of 3.7 [IQR: 1.5], overweight subjects 4.1 [IQR: 1.8], and obese subjects 3.9 [IQR: 1.67] µmol/l (P = 0.0152 for lean vs. obese). The correlations of plasma uridine concentrations with metabolic, infectious and treatment factors are shown in table 3. Plasma uridine concentrations were not found to be independently associated either with the metabolic syndrome or with HALS in HIV-1-infected patients.

**Table 3:** Correlations of serum uridine levels with metabolic, infectious and treatment factors.

	Serum uridine (µmol/l)		Serum uridine (BMI-adjusted)	
	r	P	r	P
BMI	0.1280	0.0465		
Age	0.0140	0.8205	−0.0040	0.9470
Waist circumference	0.0970	0.1218	0.0350	0.5890
WHR	−0.0010	0.9868	−0.0440	0.4920
Fat percentage	0.1630	0.0093	0.1250	0.0520
Trunk/apendicular fat ratio	− 0.0630	0.3126	-0.0690	0.2830
Fasting glucose	−0.0820	0.1950	-0.0990	0.1260
Fasting insulin	0.0420	0.5092	−0.0650	0.3170
HOMA-IR	−0.0550	0.3785	−0.0790	0.2200
MUFAs	−0.2830	< 0.0001	-0.2900	< 0.0001
PUFAs	0.3130	< 0.0001	0.3220	< 0.0001
Total cholesterol	0.2010	0.0010	0.1700	0.0080
Triglycerides	−0.0210	0.7434	−0.0490	0.4490
LDL cholesterol	0.1950	0.0019	0.1650	0.0110
HDL cholesterol	0.1440	0.0217	0.1650	0.0100
VLDL cholesterol	−0.0460	0.4703	−0.0790	0.2260
Systolic BP	0.0370	0.5550	0.0370	0.5680
Diastolic BP	0.0020	0.9775	−0.0050	0.9330
CD4 count	0.0200	0.7723	0.0150	0.8350
Plasma viral load (log_10_)	−0.0120	0.8640	0.0050	0.9470
Years of infection	−0.2230	0.0005	−0.2130	0.0010
NRTI (m)	−0.1670	0.0085	−0.1710	0.0080
NNRTI (m)	−0.0510	0.5506	−0.0300	0.7330
PI (m)	−0.1090	0.0864	−0.1130	0.0800
AZT exposure (m)	−0.0300	0.6352	−0.0220	0.7340
AZT exposure (g)	−0.0530	0.4058	−0.0480	0.4620
d4T exposure (m)	−0.1700	0.0074	−0.1760	0.0070
d4T exposure (g)	−0.1640	0.0097	−0.1720	0.0080
ddI exposure (m)	−0.1280	0.0437	−0.1190	0.0670
EFV exposure (m)	−0.1490	0.0182	−0.1500	0.0200
NVP exposure (m)	−0.0410	0.5164	−0.0410	0.5320

BMI  =  body mass index, WHR  =  waist-to-hip ratio, HOMA-r, homeostasis model assessment of insulin resistance, MUFAs  =  monounsaturated fatty acids, PUFAs  =  polyunsaturated fatty acids, LDL  =  low density lipoproteín, HDL  =  high density lipoproteín, VLDL  =  very low density lipoproteín, BP  =  blood pressure, NRTI  =  nucleoside-analogue reverse transcriptase inhibitor, NNRTI  =  non nucleoside-analogue reverse transcriptase inhibitor; PI  =  protease inhibitor; AZT  =  zidovudine, d4T  =  stavudine, ddI  =  didanosine, EFV  =  efavirenz, NVP  =  nevirapine, m  =  months; g  =  grams.

### Fat uridine concentrations

Uridine content was measured in fat biopsy samples from 19 patients (naïve: 10, no HALS: 5, HALS: 4) and 15 controls. Median uridine level in fat from HIV-1-infected patients was 6.0 [IQR: 3.67], and in controls, 2.8 [4.65] nmol/mg protein (P = 0.0118). Median fat uridine level was 6.1 [0.50], 2.6 [1.85], and 6.3 [3.4] nmol/mg protein, for naïve, non-HALS patients, and HALS patients, respectively (P = 0.3767). There was a negative correlation between plasma and fat uridine concentrations in HIV-1-infected patients (R = −0.5210, P = 0.0223), whereas such a correlation was not found for controls (R = 0.0680, P = 0.8720). Median fat uridine level was 5.5 [IQR: 4.0] nmol/mg protein for those who were currently treated with AZT or d4T, whereas it was 2.6 [IQR: 1.27] nmol/mg protein for those not treated (P = 0.5369). There were no statistically significant differences between patients with mixed HALS and pure lipoatrophic HALS with respect to fat uridine concentrations 6.80 [IQR: 0] vs. 6.55 [IQR: 0.50] nmol/mg protein, respectively, P = 0.4028). The only correlations were for EFV exposure (R = −0.4890, P = 0.0336), and for PUFAs level (R = −0.5740, P = 0.0252).

### Expression of genes encoding uridine metabolism-related enzymes and concentrative uridine transporters

**Figure 2:**
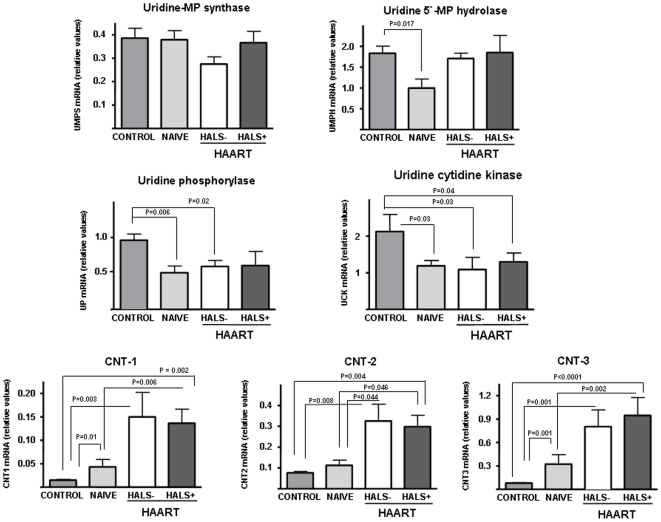
Expression of genes encoding enzymes involved in uridine metabolism and concentrative nucleoside transporters (CNT). Data from subcutaneous adipose tissue of uninfected controls and HIV-1 infected patients. Figure shows means + SEM for each specific mRNA concentration (expressed as ratio relative to HPRT mRNA) from 6-8 patients/group. P values for statistical comparisons between groups are shown when <0.05. MP  =  monophosphate. HALS-  =  No HIV-1/HAART-associated lipodystrophy syndrome. HALS+  =  HIV-1/HAART-associated lipodystrophy syndrome. HAART  =  Highly Active AntiRetroviral Therapy.

The expression of genes involved in uridine metabolism is depicted in figure 2. The expression of the transcript for UMP synthase was not significantly modified in adipose tissue from all HIV-1 infected patient groups with respect to controls, regardless of having been treated or having developed HALS or not. The expression of the gene for UMP hydrolase (also called cytosolic 5′-nucleotidase III) was slightly decreased in naïve patients but unaltered in the other groups of HIV-infected patients (Figure 2). The pattern of changes observed for uridine cytidine kinase and uridine phosphorylase (UPase) indicated a significant decrease in all groups of patients with respect to controls. There were no changes in comparisons between naïve and treated patients or in patients with or without HALS. The expression of concentrative nucleoside transporters (CNTs), potentially involved in concentrating intracellular uridine [25], showed a similar pattern of changes in the three subtypes of transporters in naïve and treated patients, in agreement with previous findings [23]. Naïve patients showed a higher expression of the mRNAs for CNTs with respect to healthy controls, which was statistically significant for CNT1 and CNT3. HAART-treated patients, either having HALS or not, showed significantly higher concentrations of expression of the three CNT mRNA subtypes with respect to controls and naïve, with distinct concentrations of statistical significance depending on the CNT subtype (Figure 2). No differences were observed for the expression of CNTs mRNAs between patients with or without HALS.

## Discussion

Our study does not suggest an association of the development of features of HALS or metabolic syndrome with plasma and fat concentrations of uridine, except for lipohypertrophy, but suggests that HIV-1 infection is associated with a significant decrease in uridine plasma concentrations. However, the number of patients with uridine <3 µmol/l was higher in patients with than in non-HALS patients. Antiretroviral therapy that included thymidine analogues did not influence uridine plasma concentrations. On the other hand, fat uridine concentrations showed an inverse correlation with plasma concentrations. This finding may be explained by an over-expression of those genes that encode concentrative nucleoside transporters, and particularly CNT3 [23], [25], as well as by under-expression of genes encoding enzymes leading to anabolic (UCK) or catabolic (UPase) uridine consumption.

However, the present results have to be viewed in the light of their inherent limitations. The first one comes from the nature of the study; this is a cross-sectional study and therefore, no causal relationships can or should be drawn. Second, for the majority of patients, uridine was measured in plasma whereas the anatomical and biological processes giving rise to HALS occur in the adipose depots. We have some measurements of uridine in fat, and although such measurements are highly reproducible and with tight variability coefficients, their reliability is difficult to ascertain [26]. In addition, the number of patients included in this substudy was low, and this may hamper the validity of our results.

The plasma uridine results are trustworthy since we were especially careful to avoid overestimations of the uridine peak, as we have observed that some unknown compounds sometimes coelute with uridine in our chromatographic separation. This fact could explain, at least in part, the values slightly higher for uridine plasma concentration reported by other groups [18], and could account for the difference between our results, which do not detect reduced uridine concentrations in treated patients, an the work by Langmann et al that, in contrast, reports significat reductions in dideoxy-NRTI treated patients [16]. We double tested enzymatically the peak of uridine by treating a second aliquot of the samples with an excess of thymidine phosphorylase from *E. coli*, which ensures that we only measured true uridine, with no interfering compounds eluting at the same retention time in the chromatogram. This may explain why plasma uridine values obtained in our patients with HALS are lower than those reported by Sutinen et al. [18] for patients with clinically evident lipoatrophy. On the other hand, the results obtained in our study compared well with previously reported uridine plasma concentrations [5], [6], [12].

We have found that HIV-1 infection is associated with significantly lower concentrations of uridine in plasma, whereas there were not differences between HIV-1-infected patients irrespective of their treatment status, but it was by the presence of lipoatrophic HALS. This may be explained by the fact that, unlike what happens in non-infected patients, there is a shift of uridine towards adipose depots and maybe other tissues. Additionally, among naïve patients, a negative correlation was found between uridine plasma concentrations and the amount of plasma HIV-1 RNA. This may suggest its consumption during the HIV-1 replicative cycle. On the other hand, alterations due to HIV-1-infection in adipose tissue from patients, i.e. reduction in the amounts of mitochondrial DNA [27], or impaired expression of genes implicated in adipocyte anabolic functions [28], have been previously reported.

Uridine supplementation has been advocated as a successful treatment for thymidine analogues-related lipoatrophy based on a short-term randomized, double-blind, placebo-controlled trial [18]. However, recent data from a randomized, double blind, placebo-controlled trial, failed to show any benefit of uridine supplementation in terms of fat gain after 48 weeks of treatment [29]. In addition, when compared with a strategy of switching from thymidine analogues to tenofovir, supplementation with uridine induced a pro-inflammatory state without showing benefit in terms of fat gain [30]. Therefore, it seems that uridine administration cannot reverse the adipose toxic effects of thymidine analogues, and may even represent an additional risk because of its potential inflammatory induction. This is not at odds with our findings concerning the positive correlation between plasma uridine concentrations and polyunsaturated fatty acid (PUFAs) concentrations. The beneficial effects of PUFAs on the cardiovascular system and on lipid profile are well known [31].

The concentrations of uridine in animal experimentation tissues are usually far in excess of the concentration of uridine in plasma [1]. Both, plasma and intracellular concentrations of uridine are regulated by the catabolic activity of uridine phosphorylase (UPase) and by transport mechanisms [9]–[11], [32]. We have found an underexpression of UPase in adipocytes which may partly justify the greater concentration of fat uridine compared to that of plasma (Figure 2). In addition, the increased expression of CNTs in HIV-1-infected patients' adipose tissue contribute to the shift of uridine into the adipose cells. The overall shift in the expression of these genes leading to induce an increase in the fat tissue pyrimidine pool fits well with a previous report suggesting a role of intracellular pyrimidine depletion associated to nucleoside analogues toxicity [15]. Although we did not see any effects in the systemic uridine concentrations, the increase of intracellular uridine in treated patients might be, in fact, a reflex of a compensatory mechanism to avoid intracellular pyrimidine depletion.

In summary, our results suggest that uridine homeostasis is profoundly disturbed by HIV-1 infection. Although plasma concentrations in HIV-1-infected patients were lower than in controls, the only additional uridine disturbance that could be documented was unrelated to antiretroviral therapy but it was to the development of pure lipoatrophic syndrome. These results are in agreement with the lack of efficacy of uridine supplements in HALS reversal as shown by ACTG 5229.

## Supporting Information

File S1 (0.03 MB DOC)
