# Supplementary material for: Uridine Metabolism in HIV-1-Infected Patients: Effect of Infection, of Antiretroviral Therapy and of HIV-1/ART-Associated Lipodystrophy Syndrome
Source: PLoS One. 2010 Nov 15;5(11):e13896. doi: 10.1371/journal.pone.0013896 (PMC2981524; doi:10.1371/journal.pone.0013896)
Supplement: File S1 — (0.03 MB DOC) [file pone.0013896.s001.doc]

**Appendix**

**Body composition measurements**

Subjects were weighed on calibrated scales after removing shoes, outdoor clothing, and other heavy items. Body mass index (BMI) was calculated by dividing the weight in kilograms by the square of the height in meters. Waist circumference was measured to the nearest millimeter using anatomical landmarks as defined for the Third National Health and Nutrition Evaluation Survey [1].

Whole body dual energy X-ray absorptiometry (DEXA) scans (Hologic QDR-4500A Hologic, INc, 590 Lincoln St, Waltham, MA 02154, USA) were conducted by a single operator in all the patients. The percentage fat at the arms, legs and central abdomen (calculated from the mass of fat versus lean and bone mass) as well as total lean body mass in kilograms was recorded.

**Definition of HALS and metabolic syndrome**

Lipoatrophy was identified by patient self-reporting and/or clinician observation of relevant changes to the face (loss of cheek and/or preauricular fat pads), arms, legs, and subcutaneous abdominal tissue, and confirmed clinically by qualitative physical examination. Lipohypertrophy was diagnosed by the presence of central adiposity which was defined by a waist-hip ratio (WHR) of > 0.90 in men, and > 0.80 in women) [1], breast enlargement in women or the appearance of cervical fat pads or supraclavicular fat accumulation [2]. Both clinical situations occurred in an isolated manner or concomitantly and any combination of them was considered to be a mixed syndrome.

Visual aspects of lipodystrophy were assessed with a lipodystrophy severity grading scale (LSGS) based on that reported by Lichtenstein et al. [2]. The degree of lipoatrophy and diffuse fat accumulation at each region was rated as absent (score of 0), mild (noticeable on close inspection, score 1), moderate readily noticeable by patient/physician, score of 2), or severe (readily noticeable to a casual observer, score of 3). The overall score was the mean of the sum of the scores given by patient and physician both for fat loss or fat accumulation. A clinical diagnosis of lipodystrophy was given to a patient with an overall score > 7. This score was chosen because it would be the LSGS of a patient with mild fat loss or mild fat accumulation at all the anatomical sites assessed (face, arms, buttocks, legs, abdomen, neck, breasts) [3]. In addition, patients with severe fat changes in at least one body location were considered as lipodystrophic [3].

In addition, facial lipoatrophy was also assessed through the scale of Fontdevila et al. [4] which established four degrees of facial lipoatrophy ranging from normal to grade three (or severe). The metabolic syndrome was defined according to the U.S. National Cholesterol Education Program (NCEP) Adult Treatment Panel III Guidelines [5] and modified as recommended in the latest American Heart Association/National Heart, Lung, and Blood Institute Scientific Statement [6].

**Biochemistry laboratory measurements**

All laboratory investigations, including samples collected for uridine assessment, were performed after a 12 h overnight fast and at least 15 minutes after the placement of a peripheral intravenous catheter. Patients were seated during blood drawing and abstained from smoking at least 15 minutes before sampling; tourniquet use was avoided when possible and if not, was maintained for less than 1 minute. All lipid measurements were performed in a Hitachi 911 system from Roche (Basel, Switzerland). Serum total cholesterol and triglycerides were measured by fully enzymatic standard method and high-density lipoprotein cholesterol by a direct method using polyethylene glycol modified enzymes (PEGME) [7]. Low density lipoprotein (LDL) cholesterol was measured after ultracentrifugation according to the Lipid Research Clinic recommended method, but utilizing the PEGME method for HDL cholesterol instead of precipitation. Very low-density lipoprotein cholesterol was measured in the fraction of d< 1006 kg/l of the ultracentrifugation [8]. Insulin resistance was estimated by the homeostasis model assessment method (HOMA-R) as the product of the fasting concentrations of plasma insulin (µunits/ml) and plasma glucose (mmol/l) divided by 22.5 [9].

**References for appendix**

1. Lemieux S, Prud'homme D, Bouchard C, Tremblay A, Despres JP. A single threshold of waist girth identifies normal-weight and overweight subjects with excess visceral adipose tissue. Am J Clin Nutr 1996; 64: 685-93.

2. Lichtenstein KA, Ward DJ, Moorman AC, Delaney KM, Young B, Palella FJ, Jr. et al. Clinical assessment of HIV-associated lipodystrophy in an ambulatory population. AIDS 2001; 15: 1389-98.

3. Asensi V, Martín-Roces E, Collazos J, et al. Association between physical and echographic fat thickness assessments and a lipodystrophy grading scale in lipodystrophic HIV patients: practical implications. AIDS Res Hum Retroviruses 2006; 22: 830-6.

4. Fontdevila J, Martinez E, Rubio-Murillo JM, Milinkovic A, Serra-Renom JM, Gatell,J. A practical classification for the surgical filling of facial lipoatrophy. Antivir Ther 2005; 10: L28.

5. Executive Summary of the Third Report of The National Cholesterol Education Program (NCEP) Expert Panel on Detection, Evaluation, and Treatment of High Blood Cholesterol in Adults (Adult Treatment Panel III). JAMA 2001; 285: 2486-97.

6. Grundy SM, Cleeman JI, Daniels SR, Donato KA, Eckel RH, Franklin BA, et al. Diagnosis and management of the metabolic syndrome: an American Heart Association/National Heart, Lung, and Blood Institute Scientific Statement. Circulation 2005; 112: 2735-52.

7. Sugiuchi H, Uji Y, Okabe,K. Direct measurement of high-density lipoprotein cholesterol in serum with polyethylene glycol-modified enzymes and sulphated alpha-cyclodextrin. Clin Chem 1995; 41: 717-23.

8. Hainline A, Karon J, Lippel K. Lipid Research Clinic Program. Manual of Laboratory Operations, Lipids and lipoprotein analysis. 2nd ed. ed. Washington,D.C.: Govt. Printing Office, 2000.

9. Matthews DR, Hosker JP, Rudenski AS, Naylor BA, Treacher DF, Turner RC. Homeostasis model assessment. Diabetologia 1985; 28: 412-19.
